# Binding Error-Induced Control States

**DOI:** 10.5334/joc.213

**Published:** 2022-04-07

**Authors:** Anna Foerster, Moritz Schiltenwolf, David Dignath, Roland Pfister

**Affiliations:** 1 University of Würzburg, DE; 2 University of Tübingen, DE

**Keywords:** binding and retrieval, cognitive control, error processing

## Abstract

Binding and retrieval of stimulus features, response features, and their attentional weighting tune cognitive processing to situational demands. The two mechanisms promote successful actions, especially in situations in which such actions depend on controlled processing. Here we explored binding and retrieval of attentional control states that follow from erroneous actions. By definition, such errors are characterized by insufficient cognitive control but at the same time, error detection has been shown to trigger corresponding adjustments to prevent future failures. We reanalyzed existing datasets and conducted a novel experiment to investigate whether error-induced control states become bound to task-relevant stimuli. Results point towards a binding and retrieval of error-induced control states; however, the effect appears to be less reliable than for binding and retrieval of specific stimulus and response features. We discuss potential implications and alternative interpretations in terms of a mediating impact of error-induced control.

## Introduction

Agents adapt behavior flexibly to current situational demands, enabling successful and efficient action control. Here we focus on two key mechanisms that promote such flexibility: Recruitment of cognitive control biases goal-directed actions over irrelevant, intrusive actions if both are conflicting (e.g., Botvinick et al., 2001) and episodic binding of behavioral episodes allows for subsequent retrieval of bound actions if other features of the episode are encountered again, creating a short-cut for efficient action control (e.g., Frings et al., 2020). Previous research has demonstrated that cognitive control and mnemonic processes are closely intertwined. More specifically, people can form a memory of previous cognitive control operations and other characteristics of the situation, which can be retrieved on a later occasion (e.g., Abrahamse et al., 2016; Crump, 2016; Egner, 2014; Schumacher & Hazeltine, 2016; Spapé & Hommel, 2008). Here, we use the term *control state* to refer to a mental set that biases attentional selection (see Egner, 2014). Evidence for retrieval of abstract control states comes from studies that reported enhanced cognitive control when a stimulus feature repeated across two consecutive trials (Dignath et al., 2019; Dignath et al., 2021; Grant et al., 2021; see also Brosowsky & Crump, 2018; Chiu & Egner, 2017; Whitehead et al., 2020). Critically, once a control state has been retrieved by a stimulus, its impact is not tied to the specific stimulus-response (S-R) codes which instantiated the control state in a previous behavioral episode nor to the currently appropriate S-R rule.

While previous research focused on cognitive control in challenging, but correct trials, the current study extends the notion of binding and retrieval of control states to erroneous actions. Theoretical accounts consider errors as a special case of conflict – while conflict during correct trials should be highest before response execution (i.e., before the correct response is selected amongst other highly active but inappropriate responses), conflict in trials with incorrect responses should be highest during and after response execution, because of the concurrent activation of the incorrect and the correct response (Foerster et al., submitted; Yeung et al., 2004). Despite these differences, errors should yield strong control states at least by the end of a behavioral episode.

### Adaptive error processing

Errors are thus critical events that signal a lack of control and therefore call for increasing control to prevent subsequent errors. These processes have been mainly studied for commission errors, i.e., situations where a response from the instructed response set was delivered that, however, violated the assigned stimulus-response rules. Already during their execution, erroneous responses can be inhibited or even cancelled swiftly (Bode & Stahl, 2014; Foerster et al., in press; Hochman et al., 2017; Rabbitt, 1978; Śmigasiewicz et al., 2020). Most important for the present research, errors also modulate subsequent actions (Rabbitt, 1966). This continued influence of errors has been predominantly studied in prolonged response times following an error relative to following a correct response (e.g., Pfister & Foerster, 2022). Such post-error slowing has been attributed to an orienting toward the error, its monitoring, as well as a reconfiguration of cognitive processing to shift toward successful responding (e.g., Crump & Logan, 2013; Jentzsch & Dudschig, 2009; Notebaert et al., 2009; Rabbitt, 1966; Wessel, 2018).

Although error processing has been an active field of research for decades, it is currently not well understood how post-error control is supported by mnemonic processes. Theoretical accounts have further proposed that only correct actions are subject to binding (Hommel, 2005), yet, recent evidence suggests a different picture. Binding of stimulus and response features does indeed take place for action slips, with the correct but not executed response being bound to task-relevant and task-irrelevant stimulus features during error commission (Foerster et al., submitted; Foerster, Moeller, et al., 2021; Foerster, Rothermund, et al., 2021). Features of the executed erroneous response still remain activated and can enter bindings with action-triggered changes in the agent’s environment. These findings characterize binding for action slips as a highly adaptive mechanism and they suggest that error-induced control states might indeed be incorporated into episodic representations as well. That is, a more conservative control state might be bound to concurrently presented stimuli so that re-encountering these stimuli would retrieve and thus reinstate the bound control state.

### Binding error-induced control states

**Figure 1:**
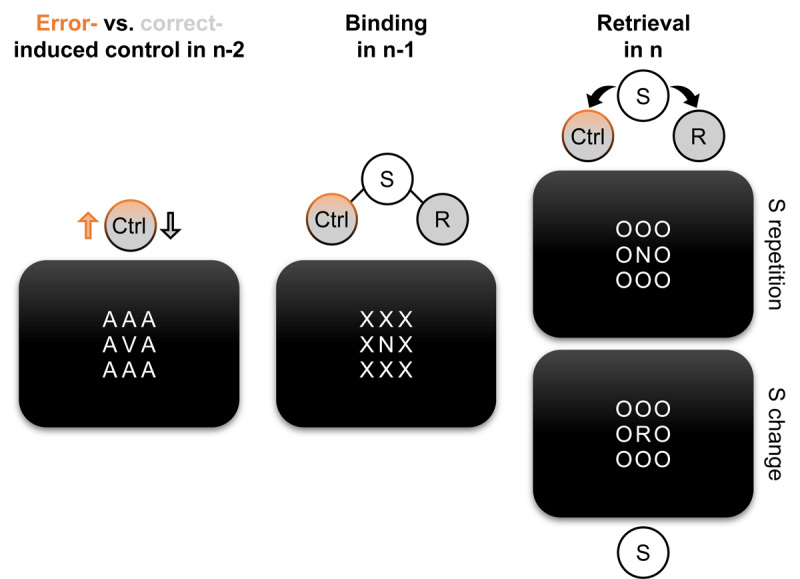
Exemplary trial sequence with proposed control state changes, and hypothesized binding and retrieval processes. We examined sequences of three trials to probe for binding and retrieval of error-induced control states. On each trial, participants responded with a left or right response to a target letter that was surrounded by irrelevant letters. The arrangement of letters corresponds to the design of the Confirmatory Experiment. Stimuli are not drawn to scale for legibility. Assumed representations of the features of an action episode are shown as circles. Binding is illustrated through lines between feature representations and retrieval through arrows. In trial n–2, a commission error (orange) should lead to increased control (Ctrl) in comparison to correct responses (grey). The resulting control state then binds to the presented stimuli (S) in trial n–1 as do features of the executed correct response (R). A repetition of the relevant stimulus in trial n is assumed to retrieve the previously bound control state and response (top), whereas retrieval does not take place for stimulus changes (bottom).

The present research approached the question of binding and retrieval of error-induced control states through sequential analyses of a speeded choice reaction task. ***Figure 1*** shows a schematic of this methodology. We hypothesized that correct and erroneous responses induce different control states that carry over to the subsequent behavioral instance (we will refer to the behavioral episode that instigates a certain control state as trial n–2). For errors, increased cognitive control manifests robustly as post-error slowing, especially for the response immediately following the error (Jentzsch & Dudschig, 2009; Li et al., 2021; Notebaert et al., 2009; Pfister & Foerster, 2022; Wessel, 2018).^1^ Empirical evidence on changes in accuracy after errors does not provide a systematic pattern although more cognitive control should lead to a post-error increase in accuracy (see the *General Discussion* for a more detailed argument). Accordingly, we focused on response times (RTs) to assess retrieval of error-induced control states. We assumed that error-induced control states are bound to relevant stimuli (S-Ctrl binding; we refer to this binding episode as trial n–1). In the same episode, the relevant stimulus is further bound to the executed correct response (S-R binding; e.g., Henson et al., 2014; Hommel, 1998). Therefore, a repetition of identical S-R pairings across two consecutive trials (i.e., trial n–1 to trial n) should facilitate performance compared to sequences with a response repetition but a target change. If error-induced control states were indeed present in trial n–1 and bound during this episode, their retrieval should slow down responding for stimulus repetition trials with errors in trial n–2 as compared to correct responses in trial n–2. We tested this hypothesis in a reanalysis of existing datasets and in a novel experiment with increased statistical power.

## Reanalysis of existing data: Error-induced binding and retrieval

The reanalyses used data from previous experiments on binding and retrieval for action slips (Reanalysis 1: Exp. 1 of Foerster, Moeller, et al., 2021; Reanalysis 2: Exp. 1 and 2 of Foerster et al., submitted). Participants in these studies had responded with keypress responses to target letters (4:2 mapping of stimuli to responses). They had a limited time-window and target letters were surrounded by noise distractors to trigger errors. The existing datasets with a 4:2 mapping had targeted S-R binding for action slips and thus provided an excellent opportunity for a first evaluation of S-Ctrl bindings. Participants did not receive feedback after a commission error in the first study. The second study replicated a condition without such feedback for half of the participants whereas the other half of the sample received feedback. Target stimuli could either repeat or change across trials, while correct responses could repeat for both target repetitions and target changes, but correct response changes were only possible for target changes. Therefore, S-R binding and retrieval effects can be assessed as the difference between correct response repetitions to relevant stimulus repetitions and changes.

Crucially, we now analyzed these differences between correct response repetitions to relevant stimulus repetitions and changes as a function of the accuracy in trial n–2. To control for any other effects of binding and retrieval from trial n–2 to n–1 and of error processing from n–1 to n, we restricted the analyses to trials with changes of the relevant stimulus and the correct response from n–2 to n–1. We also selected only trial sequences with a correct response in n–1. These necessary constraints reduced statistical power through trial and participant dropout for our reanalysis considerably. Accordingly, the reanalysis could only provide initial evidence for our hypothesis and required to be followed up by a confirmatory study using a carefully adapted experimental design.

A modulatory effect of errors versus correct responses in n–2 on the differences between correct response repetitions to relevant stimulus repetitions versus changes from n–1 to n could indeed be a consequence of binding and retrieval of a control state but there is also a plausible alternative interpretation in terms of post-error slowing. Post-error slowing increases the time interval between stimulus and response after erroneous responses relative to after correct responses (e.g., Pfister & Foerster, 2022; Rabbitt, 1966). This increased time interval might lead to weaker S-R binding (and retrieval), independent of any binding and retrieval of control states. Luckily, the selection of changes of relevant stimuli responses in trial n–1 can be expected to work against this confound. With a 4:2 mapping, correct response changes imply that participants execute the preceding erroneous response after an action slip or the previously ignored response option after a correct response. As such, a residual activation of the preceding erroneous response might facilitate responding, counteracting any control-induced slowing after errors (Foerster et al., submitted). We tested whether this pattern actually emerged by comparing RTs in trial n–1 (RT_n–1_) between a correct response or an error in trial n–2. Absent post-error slowing, or even post-error speeding would support the assumption that the predicted modulation of S-R binding and retrieval effects can be attributed to binding and retrieval of control states.

### Methods

For brevity, we will only highlight the central aspects of the methods here. More details are available in the original publications (Reanalysis 1: Exp. 1 in Foerster, Moeller, et al., 2021; Reanalysis 2: Exp. 1–2 in Foerster et al., submitted).

#### Participants

For Reanalysis 1, we used a dataset of 48 participants (33 female; 46 right-handers; age: mean = 29 years, *SD* = 11 years). The data for Reanalysis 2 came from a sample of 96 participants (80 female; 76 right-handers; age: mean = 24 years, SD = 6 years), of which half did and half did not receive feedback after a commission error.

#### Apparatus, stimuli and procedure

The studies of Reanalysis 1 and 2 were conducted in the laboratory. Participants had to respond to four target letters while ignoring surrounding irrelevant letters (similarly to the setup depicted in ***Figure 1***). These irrelevant letters were to provoke errors by increasing perceptual noise, but they did not overlap with the set of target letters and therefore also did not map to any response keys. We excluded repetitions of irrelevant letters in successive trials by design to control for any binding and retrieval related to these letters (e.g., Foerster, Rothermund, et al., 2021).

We instructed participants to respond as fast and accurately as possible. After a fixation for 750 ms, a target and irrelevant letters appeared, and participants had to respond within 600 ms. In the practice block, participants received immediate feedback about the accuracy of each response. In the following experimental blocks, participants only received immediate feedback if they did not respond but no feedback for both, correct or wrong responses. This procedure ensured that the main comparison of interest was not confounded by different visual events. An exception is the feedback condition of Reanalysis 2, where participants received immediate feedback for wrong keypresses so that this condition allowed for studying the impact of immediate error feedback on binding and retrieval. At the end of each block, we provided an overview of the performance. There were 20 blocks (including one practice block) with 56 trials per block.

#### Data treatment

We excluded the practice block as well as the first and second trial of each block. None of the participants was excluded based on their error rates. We then selected trials with a correct response (Reanalysis 1: 80.5%; Reanalysis 2: 82.8% without feedback and 84.5% with feedback) or a commission error (Reanalysis 1: 11.3%; Reanalysis 2: 8.1% without feedback and 9.5% with feedback) in trial n–2, excluding omission errors (i.e., no response before the response deadline; Reanalysis 1: 4.8% omissions, Reanalysis 2: 4.7% omissions without feedback and 4.7% omissions with feedback) and miscellaneous errors (i.e., responses with any other than the instructed keys or delivery of responses during fixation or the blank screen; Reanalysis 1: 3.4% miscellaneous errors, Reanalysis 2: 4.5% miscellaneous errors without feedback and 1.3% miscellaneous errors with feedback). To control for retrieval effects from trial n–2 to trial n–1, we only selected trials with a target change and a change of the correct response in trial n–1. We further selected trials with a correct response in trial n–1 (Reanalysis 1: 29.0%; Reanalysis 2: 21.4% without feedback and 21.5% with feedback excluded). We excluded trials with a target change and a change of correct responses in trial n. For the analysis of commission errors, we excluded omission errors (Reanalysis 1: 4.1%; Reanalysis 2: 3.5% without feedback and 3.4% with feedback) and miscellaneous errors (Reanalysis 1: 1.6%; Reanalysis 2: 1.3% without feedback and 0.9% with feedback) and computed percentage of commission errors by dividing the number of commission errors by the sum of commission errors and correct responses. For RT analyses, we only considered correct trials and excluded RTs as outliers if they deviated more than 2.5 standard deviations (*SDs*) from their cell mean (Reanalysis 1: 1.1%; Reanalysis 2: 1.1% without feedback and 1.1% with feedback). We only analyzed participants who delivered at least 5 observations in each design cell of the RT analysis (Reanalysis 1: 32; Reanalysis 2: 24 with feedback and 29 without feedback). Table S1 and Table S2 in the Supplementary Material present descriptive statistics of the number of trials for the RT analysis. For the same selection of trials, we analyzed RT_n–1_.

#### Data analyses

We analyzed RTs and percentage of commission errors in separate ANOVAs with the within-subjects factors response in n–2 (correct vs. commission error) × condition sequence from n–1 to n (target repetition | correct response repetition vs. target change | correct response repetition) and in Reanalysis 2, with the additional between-subjects factor feedback (absent vs. present). A significant three-way interaction would be followed up by separate 2 × 2 ANOVAs for the two feedback conditions. In case of a significant two-way interaction between the within-subjects factors, we scrutinized differences between condition sequences in two-tailed paired-samples *t*-tests, separately after correct and erroneous responses in n–2. We compared RT_n–1_ for correct responses and commission errors in trial n–2 in a two-tailed paired-samples *t*-test in Reanalysis 1 and in a 2 × 2 ANOVA with the factor feedback in Reanalysis 2. A significant two-way interaction in this ANOVA would be followed up by two-tailed paired-samples *t*-test for each feedback condition. We report the effect size {d_z} = {\textstyle{{M(\Delta RT)} \over {SD(\Delta RT)}}} for all *t*-tests.

In case of a significant increase of RT_n–1_ after an erroneous than after a correct response, we planned to employ a matching procedure and repeat our main analysis of RTs on the matched dataset. That is, we planned to select the trial with the lowest trial number that had an erroneous response in n–2. For this trial, we would compute a difference score of RT_n–1_ for each trial with a correct response in n–2. The correct trial with the smallest absolute difference score would be selected as match. In case of tied difference scores, we would select the correct trial with the closest trial number as a match. If there was still a tie, we would opt for the correct trial with the lowest trial number. After finding a match, we would proceed with the next higher trial number with an error in n–2 until all erroneous trials have a matching correct trial.

### Results

#### Reanalysis 1

**Figure 2:**
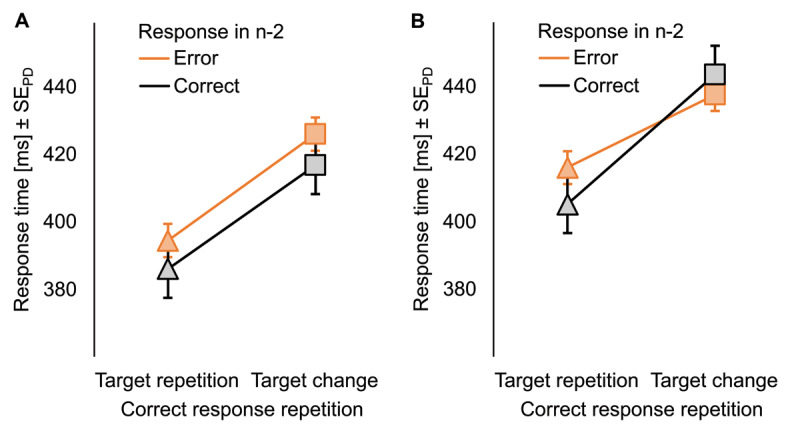
Mean response times of Reanalysis 1 and 2. Mean response times as a function of the response in n–2 (correct in black, error in bright orange) and whether correct response repetitions had to be executed for target repetitions or target changes in (A) Reanalysis 1 and (B) Reanalysis 2 for which we averaged over feedback conditions here. Error bars represent the standard error of the differences, separately computed for correct and erroneous responses in n–2.

Descriptive statistics of all measures are presented in ***Table 1***. RTs in n did not differ between correct responses and commission errors in n–2 (see ***Figure 2A***), *F*(1, 31) = 1.54, *p* = .224, η_p_^2^ = .05. Response repetitions were faster for target repetitions than for target changes, *F*(1, 31) = 31.90, *p* < .001, η_p_^2^ = .51. The interaction of both factors was not significant, *F* < 1. Commission errors were higher after a correct response than after a commission error in n–2, *F*(1, 31) = 9.34, *p* = .005, η_p_^2^ = .23, and lower if the correct response repeated in target repetition than in target change trials, *F*(1, 31) = 30.40, *p* < .001, η_p_^2^ = .50. The interaction was not significant, *F*(1, 31) = 2.83, *p* = .103, η_p_^2^ = .08. RT_n–1_ did not differ after correct responses and commission errors in n–2, *t*(31) = 1.45, *p* = .156, *d_z_* = 0.26.

**Table 1:** Descriptive data of Reanalysis 1. Means and standard deviations (in brackets) of response times in n (RT) and n–1 (RT_n–1_) and of the percentage of commission errors for each analysis cell.


RESPONSE IN N–2	CONDITION SEQUENCE IN N–1	CONDITION SEQUENCE IN N	RT [MS]	ERRORS [%]	RT_N–1_[MS]

Correct	Target change | correct response change	Target repetition | correct response repetition	386 (34)	3.2 (3.3)	455 (21)

		Target change | correct response repetition	417 (34)	13.1 (5.9)	
	
Error		Target repetition | correct response repetition	394 (63)	2.6 (5.0)	443 (49)

		Target change | correct response repetition	426 (50)	8.8 (10.9)	


#### Reanalysis 2

Descriptive statistics of all measures are presented in ***Table 2***. In RTs, there was no significant difference between correct responses and commission errors in n–2 (see ***Figure 2B***), *F* < 1. Correct response repetitions were faster if the target also repeated than if it changed, *F*(1, 51) = 98.84, *p* < .001, η_p_^2^ = .66. The main effect of feedback was not significant, *F* < 1. The two-way interactions of response in n–2 × feedback and of condition sequence from n–1 to n × feedback were not significant, *F*s < 1. However, there was the predicted two-way interaction between response in n–2 and condition sequence from n–1 to n, *F*(1, 51) = 9.01, *p* = .004, η_p_^2^ = .15, that was not further modulated by feedback, *F* < 1. Differences between condition sequences from n–1 to n were larger after a correct response in n–2, *t*(52) = 11.06, *p* < .001, *d_z_* = 1.52, than after a commission error in n–2, *t*(52) = 4.70, *p* < .001, *d_z_* = 0.65.

**Table 2:** Descriptive data of Reanalysis 2. Means and standard deviations (in brackets) of response times in n (RT) and n–1 (RT_n–1_) and of the percentage of commission errors for each analysis cell.


FEEDBACK	RESPONSE IN N–2	CONDITION SEQUENCE FROM N–2 TO N–1	CONDITION SEQUENCE FROM N–1 TO N	RT [MS]	ERRORS [%]	RT_N–1_ [MS]

Absent	Correct	Target change | correct response change	Target repetition | correct response repetition	404 (24)	4.4 (4.3)	453 (29)

			Target change | correct response repetition	441 (32)	13.1 (6.6)	
	
	Error		Target repetition | correct response repetition	417 (27)	4.5 (5.9)	444 (35)

			Target change | correct response repetition	435 (37)	13.2 (11.6)	
	
Present	Correct		Target repetition | correct response repetition	406 (27)	4.7 (4.7)	460 (27)

			Target change | correct response repetition	446 (22)	20.7 (12.3)	
	
	Error		Target repetition | correct response repetition	415 (36)	5.6 (7.2)	462 (33)

			Target change | correct response repetition	440 (44)	11.8 (10.3)	


There was a non-significant trend toward more commission errors after correct responses than after commission errors in n–2, *F*(1, 51) = 3.27, *p* = .077, η_p_^2^ = .06. Participants committed less errors for correct response repetitions if the target also repeated than if it changed, *F*(1, 51) = 68.02, *p* < .001, η_p_^2^ = .57. The main effect of feedback was not significant, *F*(1, 51) = 1.94, *p* = .170, η_p_^2^ = .04. The two-way interactions of response in n–2 × feedback and of condition sequence from n–1 to n × feedback were not significant or showed only non-significant trends, *F*s(1, 51) ≤ 3.61, *p*s ≥ .063, η_p_^2^ ≤ .07. The two-way interaction between response in n–2 and condition sequence from n–1 to n was significant, *F*(1, 51) = 5.74, *p* = .020, η_p_^2^ = .10, and further modulated by feedback, *F*(1, 51) = 5.75, *p* = .020, η_p_^2^ = .10. Without feedback, participants committed less errors for correct response repetitions if the target repeated than changed, *F*(1, 23) = 28.96, *p* < .001, η_p_^2^ = .56. The main effect of response in n–2 and the interaction were not significant, *F*s < 1. With feedback, commission errors were higher after a correct response than after a commission error in n–2, *F*(1, 28) = 6.34, *p* = .018, η_p_^2^ = .19, and correct response repetitions were less error-prone for target repetitions than target changes, *F*(1, 28) = 41.40, *p* < .001, η_p_^2^ = .60. This effect of condition sequence from n–1 to n was modulated by the response in n–2, *F*(1, 28) = 15.34, *p* = .001, η_p_^2^ = .35, with smaller effects of condition sequences from n–1 to n after commission errors in n–2, *t*(28) = 2.87, *p* = .008, *d_z_* = 0.53, than after correct responses in n–2, *t*(28) = 7.62, *p* < .001, *d_z_* = 1.41.

RT_n–1_ neither differed between correct responses and commission errors in trial n–1, *F* < 1, nor between the two feedback conditions, *F*(1, 51) = 2.70, *p* = .107, η_p_^2^ = .05. The two-way interaction was not significant either, *F*(1, 51) = 2.05, *p* = .159, η_p_^2^ = .04.

### Discussion

We reanalyzed existing datasets to arrive at a first evaluation of our hypothesis that performance benefits from S-R repetitions relative to response repetitions for a target change from trial n–1 to trial n would be reduced if a control state from an erroneous response in n–2 was bound in trial n–1 and retrieved in trial n. Despite data exclusion, we found such a modulation in the dataset with the larger sample and therefore greater statistical power (in Reanalysis 2 but not in Reanalysis 1).

This modulation was evident even though the chosen conditions ensured that there was no post-error slowing in RT_n–1_ (because we only included trial sequences for which target and correct response both changed). Therefore, we could infer that binding and retrieval of control states was effective here but the absence of post-error slowing in n–2 also entails that we do not have any indication that error processing emerged immediately after the error in n–1. We therefore conducted a reanalysis of a third dataset to probe whether error processing emerges in principle in sequences where the target and the correct response change.

## Reanalysis of existing data: Post-error slowing

Reanalysis 3 assessed post-error slowing in a dataset from an experiment with a similar setup as for Reanalysis 1-2 which, however, used a 6:3 mapping of target stimuli to response options (Exp. 3 of Foerster et al., submitted). This design introduced a condition with a change of the target and the correct response where even after an action slip, the correct response mapped to a neutral response option that neither corresponded with the preceding erroneous nor intended correct response. Therefore, we could assess post-error slowing while controlling for the impact of a preactivated response, demonstrating that error processing is indeed effective even in sequences with a target change and a change of the correct response. However, this design did not allow for a test of our main hypothesis, because target repetitions did not occur sufficiently often.

### Methods

We again only highlight central aspects of the methods that deviated from the study of Reanalysis 1 and 2. All details are provided in the original publication (Reanalysis 3: Exp. 3 in Foerster et al., submitted).

#### Participants

For Reanalysis 3, we had a dataset of 57 participants (17 female, 6 did not provide their gender; 52 right-handers; age: mean = 25 years, SD = 8 years).

#### Apparatus, stimuli and procedure

The study was conducted online. Participants had to respond to one out of six target letters on each trial by pressing one of three keys with the index, middle and ring finger of their right hand (placed on the left, down and right arrow keys). The response deadline was 700 ms. The study had one practice block with 24 trials and 19 experimental blocks with 60 trials per block.

#### Data treatment

We selected participants with at least 55% correct trials after the exclusion of the practice block as per the preregistration of the original study (20 participants excluded). We then excluded the first trial of each block from the analyses. We then selected trials with a correct response (69.9%) or a commission error (16.9%) in trial n–1 (10.9% omissions and 2.6% miscellaneous errors excluded). We only analyzed trials with a target change and a change of the correct response. For the analysis of commission errors, we excluded omission errors (9.7%) and miscellaneous errors (2.7%). For RT analyses, we only considered correct trials and excluded RTs as outliers if they deviated more than 2.5 *SDs* from their cell mean (0.7%). We only analyzed participants that delivered at least 5 observations in each design cell of the RT analysis (37 participants). Table S3 in the Supplementary Material provides descriptive statistics of the number of trials for the RT analysis.

#### Data analyses

Even without matching, there was no significant difference in RT_n–1_ after a correct compared to an erroneous response in n–2 in Reanalysis 1 and 2, given that target changes | correct response changes from n–2 to n–1 called for the execution of the preactivated, preceding erroneous response (Foerster et al., submitted). In this case, it would not be obvious from the data whether error processing emerged or whether errors were not noticed. For Reanalysis 3, we therefore assessed RTs and percentage of commission errors of target change | correct response change trials in separate repeated-measures ANOVAs comparing correct responses in n–1 with a change to the neutral response in n vs. commission errors in n–1 with a change to the neutral response in n vs. commission errors in n–1 with a change to the erroneous response in n. This comparison allowed us to assess post-error slowing for conditions that either did or did not require the execution of the preactivated, formerly erroneous response. In case of a violation of sphericity, we report Greenhouse-Geisser corrections with corresponding ɛ estimates. A significant main effect was further tested in two-tailed paired-samples *t*-tests that compared correct responses in n–1 to both commission errors in n–1 (now neutral vs. erroneous).

### Results

Descriptive statistics of all measures are presented in ***Table 3***. There was a significant difference between the three conditions in RTs, *F*(2, 72) = 27.40, *p* < .001, η_p_^2^ = .43. Correct response changes to the neutral response options were faster after a correct response than after a commission error in n–1, *t*(36) = 8.00, *p* < .001, *d_z_* = 1.31. In contrast, correct response changes to a neutral response after a correct response in n–1 did not differ from correct response changes to the erroneous response after a commission error in n–1, *t*(36) = 1.11, *p* = .276, *d_z_* = 0.18. As such, post-error slowing vanished if participants could execute the already preactivated, precedingly executed erroneous response. The corresponding ANOVA on commission errors did not return a significant effect, *F*(2, 72) = 1.66, *p* = .197, η_p_^2^ = .04.

**Table 3:** Descriptive data of Reanalysis 3. Means and standard deviations (in parentheses) of response times (RTs) and of the percentage of commission errors for each analysis cell.


RESPONSE IN N–1	CONDITION SEQUENCE IN N	RT [MS]	ERRORS [%]

Correct	Target change | correct response change to neutral	566 (22)	19.2 (6.3)

Error	Target change | correct response change to neutral	590 (24)	22.2 (11.1)

	Target change | correct response change to erroneous	570 (29)	21.3 (12.1)


### Discussion

The results of Reanalysis 3 show that error processing is still effective in sequences with a target change and a change of the required correct response because post-error slowing emerges if the correct response changes to a neutral option after an erroneous episode, suggesting that a continued activation of the erroneous response compensates for post-error slowing in trial sequences with a change to the preceding erroneous response as assessed in Reanalysis 1 and 2 (see also Foerster et al., submitted).

Still, the results on binding and retrieval of error-induced control states in Reanalysis 1 and 2 are mixed and this might be a consequence of analyzing moderate sample sizes that provided low numbers of observations for some design cells, especially for erroneous action episodes. For a conclusive test of our hypothesis, we therefore conducted a confirmatory experiment that aimed for higher statistical power by a) having a higher number of trials in a study with two sessions per participant, b) by including only participants with at least 10 observations per design cell in the analyses and c) by basing the sample size on the effect size observed in Reanalysis 2.

## Confirmatory Experiment

In the Confirmatory Experiment, we aimed at replicating the above analyses of existing datasets by scrutinizing whether error-induced control states are bound and retrieved. We conducted an online study with a tried-and-tested paradigm from the laboratory (see Exp. 2 in Foerster et al., submitted) and increased the number of trials, distributed across two sessions as the task would be too long and straining in one session.^2^ As in Reanalysis 1 and 2, we predicted that smaller performance benefits of correct response repetitions for target repetitions than target changes would emerge after an erroneous than after a correct response in trial n–2. We also evaluated the impact of correct vs. erroneous responding in trial n–2 on RT_n–1_ and controlled systematically for post-error slowing.

### Methods

We preregistered the experiment at *osf.io/stkaj* and provide data and analysis syntax in the same project on the *Open Science Framework* (*osf.io/y6rax*).

#### Participants

S-Ctrl binding and retrieval effects amounted to *d_z_* = 0.41 in Reanalysis 2, though the measures taken to improve the experimental design should increase corresponding effect sizes. We still based our sample size calculations on this conservative estimate, and 49 participants ensure to detect such an effect size with a power of 80% in a two-tailed test (α = 5%; *power.t.test* function of the *R* package *stats* version 4.0.3). We therefore opted for a sample of 50 participants due to counterbalancing. We recruited participants from Prolific who stated that they were fluent in English and had an approval rate in preceding studies of at least 70%. Participants received a reward of 7.50£ after each session. We replaced excluded participants (see *Data treatment and Data analysis*).

#### Apparatus and stimuli

The task was similar as in our preceding studies. Participants conducted the study online on their desktop devices. Participants responded to simple letter stimuli to prevent perceptual priming across experimental trials (Pashler & Baylis, 1991). Participants pressed one key for the target letters *B* and *N* and another key for the target letters *V* and *K*. They operated the *F* and *J* key with their index fingers. We counterbalanced the mapping of letter pairs to the two keys. We also introduced irrelevant letters (i.e., *O, W, X, U, Z, Y, H, A*) to increase commission errors through perceptual noise. We displayed letters in a 3 × 3 grid, of which the central letter was always from the target set while the sorrounding letter positions depicted a letter from the irrelevant letter set. Letters appeared in white font against a black background in the center of the screen.

#### Procedure

We distributed the experiment across two experimental sessions of 60 minutes each that were at least 24 hours apart to collect a high number of trials. We informed participants that they would only be invited to the second session if they performed sufficiently well in the task throughout the first session, that is if they responded correctly in at least 55% of the trials. The instructions then introduced the task explaining the mapping rule and asking participants to ignore the letters that would surround the target. We motivated participants to respond as fast and accurately as possible before each block, accompanied by a short reminder of the mapping rule. We explicitly introduced the first block as a practice block to participants.

A white fixation cross appeared against a black screen for 750 ms. The letter grid appeared afterward and remained on screen until participants responded or for a maximum of 600 ms. If participants responded correctly, the screen was blank for 1000 ms after the response. Otherwise, participants received feedback in red font for omitting any response (“Too slow!”) or pressing any other key than the correct one (commission error, i.e. left key if right key instructed and vice versa: “Wrong!”; random error, i.e. any other key than the instructed keys: “Press F or J only!”). We provided aggregated feedback about their performance at the end of each block, including mean response time and the number of the three error types. We also coded a fourth type of error, namely early or late responses during fixation, blank and feedback, but we did not give feedback for these errors.

In each experimental block, we drew targets from a list that held each target letter 14 times in a random order (56 trials in each block). A complete random sequence of target letters would have resulted in about 25% target repetitions | correct response repetitions, 25% target changes | correct response repetitions and 50% target changes | correct response changes, that is about 25% of analyzable trial sequences (target repetitions | correct response repetitions or target changes | correct response repetitions in trial n after target changes | correct response changes in trial n–1). To increase the number of analyzable trials after erroneous responses while avoiding a predictable sequence, we intervened in the selection of the target of the current trial whenever participants made a commission error in trial n–2 and had a target change | correct response change from trial n–2 to trial n–1 with a correct response in trial n–1. In this case, we forced a correct response repetition in the current trial. Whether the target repeated or changed was determined by a random draw from an array that included each of these two conditions four times. The array was shuffled whenever all elements had been drawn once. For the selection of irrelevant stimuli, we created another array holding the eight letters and randomized it in the beginning of the experiment and after each 8^th^ letter with the constraint that letters would not repeat in three successive trials to avoid that such letter repetitions would retrieve control states or responses themselves. As such, each irrelevant letter appeared seven times in each experimental block. The first block in each of the experimental session was considered practice and these blocks featured only 24 trials (six repetitions of each target letter, three repetitions of each irrelevant letter). After practice, we presented 19 experimental blocks in each session.

#### Data treatment

We excluded the practice block and then computed the percentage of correct responses in the first session for each participant. Four participants responded accurately in under 55% of the trials and were therefore not invited to the second session, excluded from any further analyses and replaced by new participants. We excluded the first two trials of each block. We then selected trials with a correct response (84.7%) or a commission error (9.4%) in trial n–2 (0.6% omissions and 5.3% miscellaneous errors excluded). We controlled for retrieval effects from trial n–2 to trial n–1 by only selecting trials with a target change and a change of the correct response in trial n–1. We further selected trials with a correct response in trial n–1 (14.7% excluded). We excluded trials with a target change and a change of correct responses in trial n.

For the analysis of the percentage of commission errors, we excluded omission errors (0.3%) and miscellaneous errors (4.3%). We only included correct trials in the RT analyses and excluded outliers as in the reanalyses above (1.3%). We excluded and replaced participants that delivered less than 10 observations in any of the design cells for the analysis of RTs in the current trial (one participant). For the same selection of trials, we analyzed RT_n–1_.

Although we did not announce this in the preregistration, we excluded and did not replace three additional participants from all analyses. After all necessary data exclusions, these participants surprisingly provided fewer sequences with a correct than an erroneous response in n–2, which is why our preregistered matching procedure did not work for them. These three participants responded relatively poorly but sufficiently accurately in the first session (on average 64.2% [*SD* = 3.9%] correct responses compared to 84.4% [*SD* = 7.7%] for the remaining participants). Their performance became worse in the second session (51.3% [*SD* = 5.5%] correct responses) while the remaining sample showed an improvement (88.1% [*SD* = 6.1%] correct responses). We did not anticipate such behavior and therefore did not include an appropriate exclusion criterion beforehand. However, the exclusion of these participants did not change any statistical decision on RTs, percentages of commission errors and RT_n–1_ before the matching procedure, while it allowed us to apply the matching procedure as intended. Table S4 in the Supplementary Material provides descriptive statistics of the number of trials for the RT analysis for the remaining participants.

#### Data analysis

The analyses were the same as for Reanalysis 1. We analyzed RTs and percentage of commission errors in separate ANOVAs with the within-subjects factors response in n–2 (correct vs. commission error) × condition sequence from n–1 to n (target repetition | correct response repetition vs. target change | correct response repetition). We scrutinized any significant two-way interactions in two-tailed paired-samples *t*-tests, testing for differences between condition sequences from n–1 to n after correct and erroneous responses in n–2, respectively. We further tested whether RT_n–1_ differed between correct responses in trial n–2 and erroneous responses in trial n–2 in a two-tailed paired-samples *t*-test. In case of a significant increase of RT_n–1_ after erroneous as compared to correct responses, we would employ the same matching procedure for RT_n–1_ as announced in Reanalysis 1 and repeat our main analysis of RTs on the matched data.

### Results

**Figure 3:**
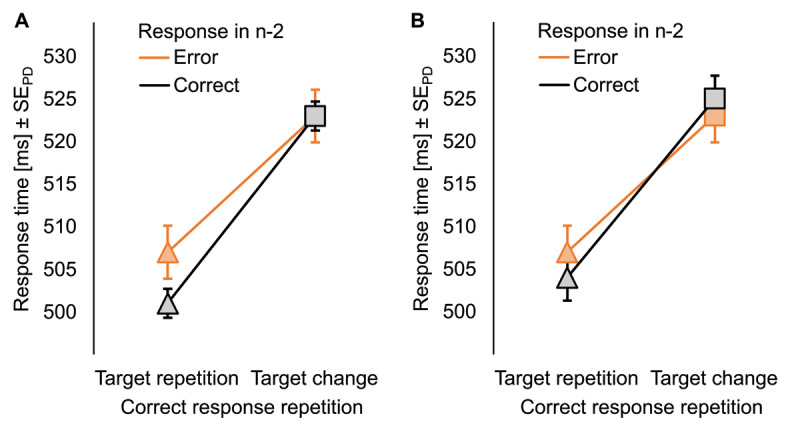
Mean response times of the Confirmatory Experiment. Mean response times as a function of the response in n–2 (correct in black, error in bright orange) and whether correct response repetitions had to be executed for target repetitions or target changes in the Confirmatory Experiment for (A) unmatched data and (B) data that was matched for effects of the response in n–2 on RT_n–1_. Error bars represent the standard error of the differences, separately computed for correct and erroneous responses in n–2.

***Table 4*** holds descriptive statistics of all measures. The main effect of response in n–2 was not significant in RTs (see ***Figure 3A***), *F*(1, 46) = 1.91, *p* = .174, η_p_^2^ = .04. Participants delivered response repetitions faster if they responded to target repetitions than to target changes, *F*(1, 46) = 84.39, *p* < .001, η_p_^2^ = .65. There was a significant interaction between both factors, *F*(1, 46) = 4.61, *p =* .037, η_p_^2^ = .09, indicating that the effect of condition sequence from n–1 to n was larger after correct responses in n–2, *t*(46) = 12.79, *p* < .001, *d_z_* = 1.87, than after errors in n–2, *t*(46) = 5.05, *p* < .001, *d_z_* = 0.74.

**Table 4:** Descriptive data of the main analyses for the Confirmatory Experiment. Means and standard deviations (in brackets) of response times in n (RT) and n–1 (RT_n–1_) and of the percentage of commission errors for each analysis cell.


RESPONSE IN N–2	CONDITION SEQUENCE FROM N–2 TO N–1	CONDITION SEQUENCE FROM N–1 TO N	RT [MS] UNMATCHED MATCHED	ERRORS [%]	RT_N–1_ [MS]: UNMATCHED MATCHED

Correct	Target change | correct response change	Target repetition | correct response repetition	501 (39) 504 (39)	6.1 (3.9)	521 (35) 531 (36)

		Target change | correct response repetition	523 (36) 525 (36)	14.5 (7.3)	
	
Error		Target repetition | correct response repetition	507 (39) 507 (39)	5.0 (4.9)	530 (36) 530 (36)

		Target change | correct response repetition	523 (35) 523 (35)	12.0 (8.2)	


The percentage of commission errors was higher after a correct response than after a commission error in n–2, *F*(1, 46) = 7.39, *p* = .009, η_p_^2^ = .14, and lower if the correct response repeated in target repetition than in target change trials, *F*(1, 46) = 79.59, *p* < .001, η_p_^2^ = .63. The interaction was not significant, *F*(1, 46) = 1.62, *p* = .209, η_p_^2^ = .03.

Post-error slowing emerged in RT_n–1_, *t*(46) = 3.66, *p* = .001, *d_z_* = 0.53. After matching RT_n–1_ for erroneous and correct responses in n–2, *t*(46) = 1.61, *p* = .115, *d_z_* = 0.23, the main effect of response in n–2 had still no significant impact on RTs in n (see ***Figure 3B***), *F* < 1. Response repetitions to target repetitions remained faster than to target changes, *F*(1, 46) = 53.77, *p* < .001, η_p_^2^ = .54. The two-way interaction was not significant anymore, *F*(1, 46) = 2.52, *p* = .119, η_p_^2^ = .05.

#### Explorative analyses

In a two-tailed Pearson correlation, we explored the relation of the interaction effect of response in n–2 and condition sequence from n–1 to n in RT with post-error slowing in RT_n–1_. The correlation was not significant, *r*(47) = .10, *t*(45) = 0.67, *p* = .507. Further, we computed an ANOVA with the within-subjects factors dataset (unmatched vs. matched) × response in n–2 (correct vs. commission error) × condition sequence from n–1 to n (target repetition | correct response repetition vs. target change | correct response repetition). Importantly, neither the interaction of dataset and condition sequence from n–1 to n, nor the three-way interaction were significant, *F*s < 1. Accordingly, a two-tailed paired-samples *t*-test did not reveal significant differences in the modulation of condition sequence from n–1 to n by response in n–2 in RT between the unmatched (*M* = 6 ms, *SD* = 20 ms) and the matched datasets (*M* = 5 ms, *SD* = 20 ms), *t*(46) = 0.62, *p* = .540, *d_z_* = 0.09. Matching led to an average data loss of 80% (*SD* = 11%) of the trials with a correct response in n–2.

Finally, we performed a median split of RT_n–1_ for each participant, separately for each design cell (response in n–2 × condition sequence from n–1 to n) and then computed an ANOVA on RT with the within-subjects factors RT_n–1_ length (short vs. long) × response in n–2 (correct vs. commission error) × condition sequence from n–1 to n (target repetition | correct response repetition vs. target change | correct response repetition).^3^ All participants of the main analysis provided at least five observations for each of the eight design cells. ***Table 5*** holds descriptive statistics of this analysis. RT was prolonged after long relative to short RT_n–1_, *F*(1, 46) = 113.02, *p* < .001, η_p_^2^ = .71. The main effect of response n–2 was not significant, *F*(1, 46) = 1.53, *p =* .222, η_p_^2^ = .03. Correct response repetitions were faster for target repetitions than changes, *F*(1, 46) = 80.54, *p* < .001, η_p_^2^ = .64. The interaction between RT_n–1_ length and response n–2 was not significant, *F* < 1, and there was only a non-significant trend toward an interaction between RT_n–1_ length and condition sequence from n–1 to n, *F*(1, 46) = 3.29, *p =* .076, η_p_^2^ = .07, pointing to somewhat larger differences between response repetitions to target repetitions than target changes for short RT_n–1_ than long RT_n–1_. The two-way interaction of response n–2 and condition sequence from n–1 to n was significant, *F*(1, 33) = 4.70, *p =* .035, η_p_^2^ = .09, indicating that the effect of condition sequence from n–1 to n was larger after correct responses in n–2, *t*(46) = 12.82, *p* < .001, *d_z_* = 1.87, than after errors in n–2, *t*(46) = 4.84, *p* < .001, *d_z_* = 0.71. The three-way interaction was not significant, *F*(1, 46) = 1.30, *p =* .260, η_p_^2^ = .03. As the modulations of condition sequence from n–1 to n by response in n–2 and by RT_n–1_ length were of comparable size, we further explored the impact of these two variables on RT_n–1_ in a further ANOVA. Both, long compared to short RT_n–1_, *F*(1, 46) = 2262.18, *p* < .001, η_p_^2^ = .98, and erroneous compared to correct responses in n–2 prolonged RT_n–1_, *F*(1, 46) = 10.73, *p* = .002, η_p_^2^ = .19, without an interaction between the two factors, *F* < 1. Comparing the relevant effect sizes between the analysis of RT_n–1_ and RT renders the alternative explanation of a covert modulation of the effect of condition sequence from n–1 to n by response in n–2 through differences in RT_n–1_ unlikely: The impact of RT_n–1_ length on RT_n–1_ was η_p_^2^ = .98, and η_p_^2^ = .07 on the difference between condition sequences from n–1 to n, whereas the impact of response in n–2 on RT_n–1_ was η_p_^2^ = .19, and η_p_^2^ = .09 on the difference between condition sequences from n–1 to n.

**Table 5:** Descriptive data of explorative analyses for the Confirmatory Experiment. Means and standard deviations (in brackets) of response times in n (RT) and n–1 (RT_n–1_) and of the percentage of commission errors for each analysis cell.


RT_N–1_	RESPONSE IN N–2	CONDITION SEQUENCE FROM N–2 TO N–1	CONDITION SEQUENCE FROM N–1 TO N	RT [MS]	RT_N–1_ [MS]

Short	Correct	Target change | correct response change	Target repetition | correct response repetition	488 (39)	472 (36)

			Target change | correct response repetition	515 (36)	
	
	Error		Target repetition | correct response repetition	496 (39)	480 (37)

			Target change | correct response repetition	512 (38)	

Long	Correct	Target change | correct response change	Target repetition | correct response repetition	514 (39)	569 (35)

			Target change | correct response repetition	532 (37)	
	
	Error		Target repetition | correct response repetition	519 (44)	579 (38)

			Target change | correct response repetition	533 (41)	


### Discussion

We hypothesized that error-induced control states could be bound to and retrieved by stimuli. We found supportive evidence for this claim because the benefit of repeating a correct response to a target repetition compared to a target change from trial n–1 to trial n was larger if the response in trial n–2 was correct than erroneous. In contrast to the former reanalyses, we found post-error slowing in trial n–1, which might instead explain this modulation through an impact of the temporal distance between S and R features on the strength of S-R bindings. After controlling for this confound through matching trials with a correct response in n–2 to trials with an erroneous response in n–2 for RT_n–1_, the hypothesized modulation was not significant anymore.

However, a close look at the descriptive statistics nourishes doubts on whether the hypothesized confound is real. For one, if post-error slowing indeed diminished S-R binding in n–1, the impact of condition sequence from n–1 to n should have been smaller in the matched than in the unmatched dataset, but we did not find any meaningful differences in post-hoc tests. Second, a close look at the modulation of the effect of condition sequence from n–1 to n through the response in n–2 revealed that these modulations were numerically very similar in the unmatched and matched datasets and did not differ significantly in a direct comparison. The modulation in the unmatched data also did not show a statistically meaningful correlation with post-error slowing in n–1. Finally, a very powerful (and arbitrary) impact of a median split of RT_n–1_ on RT_n–1_ itself, resulted only in a non-significant trend toward a modulation of the difference between target repetitions and target changes that was descriptively even smaller than the significant modulation of that difference by the response in n–2. Considering that the effect of the response in n–2 on RT_n–1_ was five times smaller than the effect of the median split, renders differences in RT_n–1_ unlikely as a mediator for the interaction between response n–2 and condition sequence from n–1 to n in RT. Taken together, the data pattern suggests that matching probably did not control for a confound but only produced a considerable drop in power by excluding a great share of post-correct trials from the analyses.

We introduced a regularity in stimulus and response relations in the two trials following each commission error as one measure to increase the number of observations for these sequences, because we had expected commission errors to occur only rarely. Participants might have picked up that if a commission error was followed by a target change and a correct response change that they also executed correctly, the upcoming trial would require a repetition of the current correct response. This prediction of the upcoming response and its preparation before stimulus onset might have facilitated responding in general, which would result in faster and more accurate responses after an erroneous than after a correct response in n–2. Such a pattern indeed emerged in the analysis of percentages of commission errors but not in RTs. However, a similar increase in accuracy was also present in Reanalysis 1 and 2 where the experimental procedure did not introduce such a regularity.

## General discussion

The current study combined evidence from multiple existing datasets and one novel experiment. We found empirical evidence for binding and retrieval of error-induced control states whenever power was comparably high. In Reanalysis 2, the hypothesized effect emerged even though potentially confounding influences in terms of post-error slowing in the binding instance of error-induced control states were absent. In the Confirmatory Experiment, we observed both effects instead. Controlling for post-error slowing led to great data loss so that only a descriptive pattern in support of S-Ctrl binding and retrieval remained, though numerically almost identical to the unmatched data. Accordingly, errors seem to shape action control both through binding of responses and control states, however, the impact of compounds with error-induced control states appears to be smaller and less reliable than the impact of compounds with specific responses (Foerster et al., submitted; Foerster, Moeller, et al., 2021; Foerster, Rothermund, et al., 2021). We can only speculate about the reason for these differences. For one, responses carry distinct category features (e.g., left vs. right) but also efferent activity (e.g., touching the key). Therefore, responses offer more concrete and distinct features than error-induced control states (Egner, 2014), and such concrete features might enter bindings more easily. In contrast, decay of bindings would point to stronger binding and retrieval of control states than responses, because S-R bindings seem to decay more rapidly (Hommel & Colzato, 2004; Hommel & Frings, 2020) than S-Ctrl bindings, at least in correct action episodes (Schiltenwolf et al., submitted). Third, even if a stimulus retrieves a response and a control state in parallel, the response might sometimes be executed rapidly even before the control state takes full effect. Finally, from a methodological perspective, data selection is even more rigid when it comes to studying S-Ctrl than S-R binding, requiring more elaborate data collection to compensate for the drop in statistical power due to data exclusion.

We hypothesized that the repetition of a stimulus would independently retrieve both, a bound control state and a response, leading to smaller benefits of repeating a response upon target repetition than upon target change after an error because of a more conservative control state. More speculatively, interference through concurrent binding of both features to the same stimulus in trial n–1 would predict a similar data pattern. Relatedly, stimuli, responses and control states could be integrated into a single compound rather than into two binary bindings. However, at least responses seem to enter separate bindings with stimuli and effects at the same time without any indication of bindings between the latter two features or all three features (Moeller et al., 2019). Finally, the parallel retrieval processes of the two features in trial n might interfere with each other, offering a third alternative explanation for the hypothesized interaction.

In addition to these alternative explanations that all assume binding and retrieval of compounds of stimuli and error-induced control states, the hypothesized interaction could potentially stem from lingering (instead of bound and retrieved) error-induced control. First, having a more conservative response criterion in the episode after an error might hamper the creation of short-cuts in action control through binding of stimuli and responses, also diminishing retrieval upon stimulus repetition. Relatedly, recent evidence points to diminished binding of task control to stimuli if agents are inattentive during the binding episode (Whitehead et al., 2021). This issue could be solved by employing designs where effects of error-induced control retrieval from a stimulus can be assessed separately from effects of response retrieval from the stimulus (i.e., by manipulating sequences of *irrelevant* stimuli, where each sequence appears equally often with response repetitions and changes). A downside of such a procedure is that binding effects with irrelevant stimuli are usually smaller than with relevant stimuli, calling for even bigger sample sizes and longer experimental sessions. Second, lingering increased control even in the second episode after an error might disturb retrieval of responses from stimuli also producing the hypothesized interaction. This assumption also predicts post-error slowing in this episode, however, we did not find significant main effects of response in n–2 (error vs. correct) on RT in n in Reanalysis 1 and 2 or the Confirmatory Experiment. But we did find a significant reduction of the percentage of commission errors in n after an error relative to after a correct response in n–2 in Reanalysis 1, in the Confirmatory Experiment, and a descriptive pattern in Reanalysis 2. This pattern of results could point to a lingering conservative response threshold after errors, which might produce our hypothesized interaction without any error-induced binding and retrieval. This issue could be resolved in paradigms that introduce intervening episodes between binding and retrieval instances (e.g., Whitehead et al., 2020), however, effects in these paradigms might relate to slightly different mnemonic processes, relying on retrieval of bindings from long-term storage rather than from working memory.^4^

Although changes in control states after the commission of an error are supposed to shift responding to a more conservative threshold, there is ample evidence for slowing but not for an increase in accuracy after an error, and this issue has been attributed to a succession of maladaptive and adaptive error processing (Wessel, 2018). That is, after an error, agents first orient toward the error, processing it thoroughly at the expense of other information. Then adaptations in cognitive control kick in to tune actions toward success. The first step predicts a drop in accuracy, the second step an increase. Dual task paradigms differentiated maladaptive and adaptive processing successfully in response times (Jentzsch & Dudschig, 2009; Steinhauser et al., 2017). Accuracy was also lower shortly after an error relative to after a correct response. However, this drop in accuracy only got smaller or vanished but did not reverse at a timepoint where only adaptive, not maladaptive processes should have been effective. Crucially, these results were obtained in sequential analyses of two successive trials. In the current study, we found a significant increase in accuracy after an error in n–2 relative to after a correct response in n–2 in Reanalysis 1, in the Confirmatory Experiment, and a descriptive pattern in Reanalysis 2. Although these results await a thorough empirical investigation, they point to a blind spot in the error literature. While adaptive mechanisms might have immediate consequences on the speed of response selection, they might take longer to have a beneficial effect on accuracy. That is, errors might be more likely to appear in chunks of two or more consecutive errors but might then promote a shift toward more accurate responding. Studying sequences of multiple responses in the realm of errors might offer novel insight for the temporal development of accuracy after an error (Pfister & Foerster, 2022). The current study also suggests that binding and retrieval extends the impact of error-induced control states on response times beyond the first action after an error where it has been investigated in most studies. Similar steps have been taken to study the impact of mistakes due to confusion of tasks sets on the representation strength of the appropriate task (Moretti et al., 2021). Future research should take an even broader temporal perspective to explore the impact of contingencies of stimuli and error-induced control states. Episodic bindings might contribute to the detection and incorporation of these contingencies, promoting the retrieval of contingent control states upon stimulus presentation as it has been proposed for adaptation to conflicting stimuli (Egner, 2014; see also Bugg & Crump, 2012).

## Conclusion

Errors recruit a variety of control processes tuned toward goal-directed behavior. Stimuli, responses and effects enter bindings that promote successful responding in the future through retrieving these bindings upon repetition of one of the features of the compound. An upregulation of cognitive control after error commission further leads to more conservative responding irrespective of the specific stimuli and actions. The current study suggests that error-induced control states can also be bound to, and retrieved by stimuli, allowing agents to deploy cognitive control specifically for the situation at hand. Whether the observed effects instead point to a more indirect role of error-induced control states on binding and retrieval of stimuli and responses awaits further examination.

## Data Accessibility Statement

The analysis syntax (*osf.io/y6rax*) and the data (Reanalysis 1: “Exp1_Raw.dat” in *osf.io/3at7x*; Reanalysis 2: *osf.io/u837c*; Reanalysis 3: *osf.io/t25nb*, Confirmatory Experiment: *osf.io/y6rax*) are publicly available via the provided links.

## Additional File

The additional file for this article can be found as follows:

10.5334/joc.213.s1 Supplementary Material. Tables S1 to S4.

## Footnotes

Post-error slowing occurs at least for repetitions of the correct response and for changes of the correct response to a neutral response that does not represent the erroneous response itself. However, post-error slowing and sustained activation of the erroneous response cancel each other out if the erroneous response is required in the trial following error commission (Foerster et al., submitted). We will capitalize on this empirical pattern during the following analyses.

We employed a 4:2 mapping as in Reanalysis 1 and 2 although a 6:3 mapping would have allowed us to test for post-error slowing in RT_n–1_ for correct response changes to a neutral response option as in Reanalysis 3. More response options would have significantly reduced the share of trial sequences with a correct response repetition that were necessary to test our main hypothesis. Considering the already substantial length of the task, we decided against further increasing the trial numbers.

We thank the reviewer Luca Moretti for proposing this analysis.

We attempted to reanalyze the data of Whitehead et al. (2020) but cell observations were too low for a meaningful test of our hypothesis. We thank the reviewers Senne Braem and Shengjie Xu for stimulating this discussion and for proposing this reanalysis.
