# Supplementary material for: Binding Error-Induced Control States
Source: J Cogn. 2022 Apr 7;5(1):24. doi: 10.5334/joc.213 (PMC9400645; doi:10.5334/joc.213)
Supplement: Supplementary Material. — Tables S1 to S4. [file joc-5-1-213-s1.pdf]

Supplementary Material

Table S1
Number of trials for the RT analysis of Reanalysis 1.

Response in n-2	Condition sequence in n-1	Condition sequence in n	*M*	*SD*	Range
Correct	Target change | correct response change	Target repetition | correct response repetition	125	23	[26-125]
		Target change | correct response repetition	98	22	[13-98]
Error		Target repetition | correct response repetition	10	4	[5-19]
		Target change | correct response repetition	9	3	[5-16]

Note. Means (*M*), standard deviations (*SD*) and the range of the number of trials for each cell in the RT analysis.

Table S2
Number of trials for the RT analysis of Reanalysis 2.

Feeback	Response in n-2	Condition sequence in n-1	Condition sequence in n	*M*	*SD*	Range
Absent	Correct	Target change | correct response change	Target repetition | correct response repetition	70	20	[35-100]
			Target change | correct response repetition	62	19	[23-92]
	Error		Target repetition | correct response repetition	9	2	[6-14]
			Target change | correct response repetition	8	2	[5-15]
Present	Correct		Target repetition | correct response repetition	70	20	[17-107]
			Target change | correct response repetition	57	20	[13-86]
	Error		Target repetition | correct response repetition	10	4	[5-18]
			Target change | correct response repetition	8	2	[5-12]

Note. Means (*M*), standard deviations (*SD*) and the range of the number of trials for each cell in the RT analysis.

Table S3
Number of trials for the RT analysis of Reanalysis 3.

Response in n-2	Condition sequence in n	*M*	*SD*	Range
Correct	Target change | correct response change to neutral	382	81	[229-511]
Error	Target change | correct response change to neutral	39	12	[5-62]
	Target change | correct response change to erroneous	40	12	[8-63]

Note. Means (*M*), standard deviations (*SD*) and the range of the number of trials for each cell in the RT analysis.

Table S4
Number of trials for the RT analysis of the Confirmatory Experiment.

Response in n-2	Condition sequence in n-1	Condition sequence in n	*M*	*SD*	Range
Correct	Target change | correct response change	Target repetition | correct response repetition	166	35	[68-246]
		Target change | correct response repetition	161	38	[66-222]
Error		Target repetition | correct response repetition	32	11	[14-53]
		Target change | correct response repetition	28	9	[15-47]

Note. Means (*M*), standard deviations (*SD*) and the range of the number of trials for each cell in the RT analysis.
